# Supplementary material for: Measuring Structural Changes in Cytochrome c under Crowded Conditions Using In Vitro and In Silico Approaches
Source: Polymers (Basel). 2022 Nov 9;14(22):4808. doi: 10.3390/polym14224808 (PMC9692323; doi:10.3390/polym14224808)
Supplement: Supplementary file 1 [file polymers-14-04808-s001.zip › polymers-1717292-supplementary.pdf]

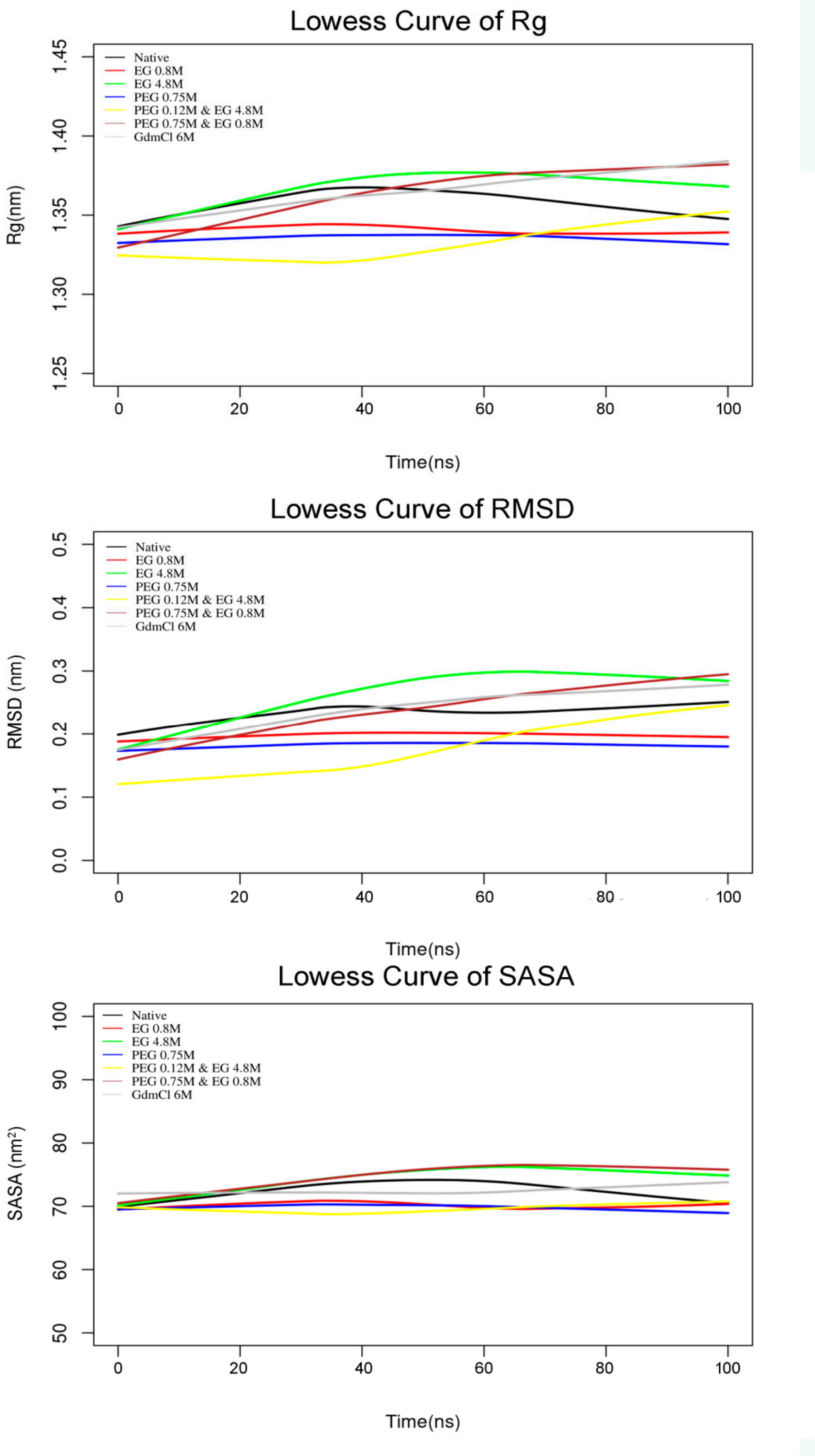

Figure S1: The Lowess Curve Plots for Rg, RMSD, and SASA for the **Figure 3.**

The apparent noise in **Figure 3** is due to the high number (7) of plots superimposed for the sake of comparison. Keeping separate plots for comparing the effect of solvent on cyt *c* in comparison to the native structure would result in higher number of figures in the main text. Therefore, we presented the figures as they are in the manuscript. However, as per the suggestion and for the better understanding of the readers the supplementary figures (**Figure S1) is** provided in the form of Lowess Curve Plots for Rg, RMSD, and SASA. The lowess plots are helpful in observing the trends on line plots having higher number of variables plotted as function of time. We hope that we are able to fulfill the requirement of the reviewer.
